# Stem cell therapy to protect and repair the developing brain: a review of mechanisms of action of cord blood and amnion epithelial derived cells

**DOI:** 10.3389/fnins.2013.00194

**Published:** 2013-10-24

**Authors:** Margie Castillo-Melendez, Tamara Yawno, Graham Jenkin, Suzanne L. Miller

**Affiliations:** ^1^The Ritchie Centre, Monash Institute of Medical Research, Monash UniversityClayton, VIC, Australia; ^2^Department of Obstetrics and Gynaecology, Monash UniversityClayton, VIC, Australia

**Keywords:** Perinatal brain injury, stem cells, umbilical cord blood, amnion epithelial cells, cerebral palsy, clinical trials, hypoxia/ischemia, inflammation

## Abstract

In the research, clinical, and wider community there is great interest in the use of stem cells to reduce the progression, or indeed repair brain injury. Perinatal brain injury may result from acute or chronic insults sustained during fetal development, during the process of birth, or in the newborn period. The most readily identifiable outcome of perinatal brain injury is cerebral palsy, however, this is just one consequence in a spectrum of mild to severe neurological deficits. As we review, there are now clinical trials taking place worldwide targeting cerebral palsy with stem cell therapies. It will likely be many years before strong evidence-based results emerge from these trials. With such trials underway, it is both appropriate and timely to address the physiological basis for the efficacy of stem-like cells in preventing damage to, or regenerating, the newborn brain. Appropriate experimental animal models are best placed to deliver this information. Cell availability, the potential for immunological rejection, ethical, and logistical considerations, together with the propensity for native cells to form teratomas, make it unlikely that embryonic or fetal stem cells will be practical. Fortunately, these issues do not pertain to the use of human amnion epithelial cells (hAECs), or umbilical cord blood (UCB) stem cells that are readily and economically obtained from the placenta and umbilical cord discarded at birth. These cells have the potential for transplantation to the newborn where brain injury is diagnosed or even suspected. We will explore the novel characteristics of hAECs and undifferentiated UCB cells, as well as UCB-derived endothelial progenitor cells (EPCs) and mesenchymal stem cells (MSCs), and how immunomodulation and anti-inflammatory properties are principal mechanisms of action that are common to these cells, and which in turn may ameliorate the cerebral hypoxia and inflammation that are final pathways in the pathogenesis of perinatal brain injury.

## Perinatal hypoxic-ischemic brain injury

The neurological consequences of a severe hypoxic-ischemic insult in the human brain at term birth are often dire, with resultant hypoxic ischemic encephalopathy (HIE) a principal cause of newborn death or permanent neurological disability, such as cerebral palsy (Johnston et al., 2011; Volpe, 2012). At risk neonates include those who show signs of fetal distress prior to delivery, have abnormal Apgar scores and require resuscitation at birth, and show specific neurological abnormalities during the first 24 h after delivery (Perlman, 2004). Although the underlying causes and exact timing of brain injury in HIE infants are not always known, there may be a principal severe intrapartum hypoxic-ischemic insult at birth caused by placental abruption, umbilical cord prolapse, or prolonged labor, but there may also be precipitating antenatal risk factors (such as preeclampsia, antenatal bleeding, or intrauterine growth restriction) (Itoo et al., 2003; Paolo, 2012).

The progression from hypoxic-ischemic insult through to mass cell death within the perinatal brain has been extensively examined and reviewed (Volpe, 2001a, 2012; Ferriero, 2004; Gunn and Bennet, 2009). It may be described as a series of phases beginning with the primary insult and energy depletion through to subsequent reperfusion-induced cell death cascades. Both human neuroimaging and experimental animal studies demonstrate that damage to the developing brain evolves over days and possibly weeks (Ferriero, 2004). In human infants, neuronal cell death occurs in a series of chronological phases after the original hypoxic-ischemic insult (Penrice and Nussey, 1992; Azzopardi and Edwards, 2010), a finding that has been explored in greater detail in animal models of perinatal brain injury (Berger and Garnier, 2000; Gunn and Bennet, 2009). Primary neuronal death, in response to the insult, occurs due to cellular hypoxia and exhaustion of high-energy stores (primary energy failure). After return of cerebral perfusion and oxygenation, the hypoxia-induced cytotoxic oedema and accumulation of excitatory amino acids often resolves, together with recovery of the depressed metabolic state of the brain. However, cerebral oxidative metabolism may deteriorate in the hours following an asphyxic period, and this phase may last for many days. This secondary energy failure can be marked by the onset of seizures, further oedema, accumulation of excitotoxins and cytotoxic inflammatory substances, failure of cerebral mitochondrial activity and ultimately cell death (Gunn and Thoresen, 2006). The duration and severity of this second, delayed wave of cerebral compromise is closely associated with the degree of neurodevelopmental compromise at 1 year of age (Roth et al., 1992). By analogy with events that occur in the adult brain after stroke or traumatic brain injury, the damage arising during the “recovery” phase is thought to be due to vascular paralysis, enhancement of inflammatory processes, free radical formation, and breakdown of the blood-brain barrier. Similarly in the developing brain, we and others have shown that increased free radical formation (Miller et al., 2005) and oedema (Bennet et al., 2007) occur in the fetal sheep brain model of brief acute *in utero* asphyxia, suggesting that the coupling of oxidative metabolism, oxygen supply, and cerebral blood flow remain disturbed for some hours after such events.

Presently, the only treatment available for babies diagnosed with HIE soon after birth is to initiate hypothermia therapy. Hypothermia as a therapeutic intervention has been extensively investigated in human newborns (Gunn et al., 1998; Shankaran et al., 2005; Simbruner et al., 2010; Higgins et al., 2011), where hypothermia, after severe hypoxia-ischemia at birth, lowers the incidence of death or major disability, resulting in significant improvements in babies with moderate, but not severe, HIE (Shankaran et al., 2005; Higgins et al., 2011). The principal mechanisms of hypothermia-induced neuroprotection are likely to be multi-modal, with hypothermia functioning to reduce brain perfusion and metabolism, decrease secondary energy failure and oxidative stress leading to recovery of cerebral oxidative metabolism, and a subsequent reduction in programmed cell death (Katz et al., 2004). However, despite demonstrated efficacy, when hypothermia is effectively applied 40–50% of infants will still die or suffer significant neurologic disability following treatment (Edwards et al., 2010; Massaro et al., 2013). Furthermore, variations currently exist in the mode of administration of therapeutic hypothermia (Harris et al., 2013) and to be effective, hypothermia to treat HIE must commence within 6 h after birth, indicative that the “window of opportunity” to reduce the progression of brain injury is limited to the immediate hours after the insult (Vannucci and Perlman, 1997; Gunn et al., 2005; Higgins et al., 2011). This is in contrast to the adult brain, where it has been shown that treatment options extend over several hours post insult and possibly days following a severe hypoxic-ischemic event (Horn and Schlote, 1992). However, any therapeutic intervention that exists to limit the degree of newborn brain injury is extremely encouraging and provides a basis and the impetus to further refine and develop new or adjunct neuroprotective treatments. Therapies that can complement and provide additive benefit to hypothermia must be considered where the principal aim is to prevent or reduce the progression of mass programmed cell death. Alternatively, where a lack of perinatal brain injury diagnosis or other logistical factors, such as availability of tertiary care, preclude therapies within the hours that comprise the window of opportunity, we must look toward alternative strategies such as cell based therapies that could provide regenerative and repair capacity within the young brain.

It should also be considered that while term hypoxic-ischemic brain injury, and subsequent HIE, is a condition that is readily identifiable and therefore amenable to treatment, there are other significant chronic or acute causal factors that contribute to perinatal brain injury and neurodevelopmental deficits. Most notably, in infants born preterm and in infants exposed to intrauterine inflammation (chorioamnionitis), white matter brain injury, which often manifests as periventricular leukomalacia, is the most common form of brain injury (Volpe, 2001b; Yoon et al., 2003). In turn, periventricular white matter injury is the predominant neuropathology observed in children with cerebral palsy (Leviton and Paneth, 1990). Defining the time of onset and evolution of white matter damage to the preterm brain is considerably more challenging than delineating injury progression in response to term hypoxia-ischemia. However, it is known that hypoxia-ischemia and upregulation of inflammatory processes are final common pathways contributing to periventricular white matter brain injury in the newborn (Volpe, 2001b; Yoon et al., 2003; Titomanlio et al., 2011).

## Stem cell therapy for perinatal brain injury

Experimental clinical and animal studies have begun to elucidate the utility of stem cell-based therapies to prevent or repair perinatal brain injury. Predominantly, these cells have been derived either from neuronal embryonic or adult tissue (neural stem cells), or from non-neural origin such as those isolated from bone marrow and umbilical cord. Neural stem cells are self-renewing and give rise to neurons, astrocytes, and oligodendrocytes. Experimentally, these cells appeared to hold great promise for neural repair after injury, including in perinatal hypoxic ischemic brain injury (Felling et al., 2006; Daadi et al., 2013), however, the therapeutic benefits of transplantation of neural stem cells have not previously been convincingly shown (Andressen, 2013). Neural stem cells are difficult to harvest, lack homogeneity, and are found in very low numbers in the central nervous system (Sommer and Rao, 2002). Stem cell therapies offer promising treatment potential, with the exciting prospect that stem cells may be able to act via a variety of diverse actions at different phases of brain injury progression. However, in addition to consideration of their efficacy, it is important to reflect on ethical concerns, accessibility, and abundance of stem cells for clinical applications. Accordingly, in this paper we will review stem cell-based therapies using human amnion epithelial cells (hAECs) and umbilical cord blood (UCB) derived cells; each with the potential to interrupt both the evolutionary progression of perinatal brain injury when the timing of the adverse insult is known, or to provide regenerative capacity when the insult may have occurred *in utero* and may, therefore, present as more advanced neuropathology. Both hAECs and UCB can be easily and economically obtained at human birth, from tissues that are otherwise discarded. Accordingly, with the prospect that each of these cell lineages display independent protective and reparative properties, we raise the intriguing possibility for autologous neuroprotective therapies for the newborn brain in babies identified at-risk of brain injury.

## Human amnion epithelial cells (hAECs)

Over the past decade there has been growing interest in the reparative properties and potential uses of hAECs in treating organ injury, including the rescue of adult neurodegenerative or acute-onset brain disorders (Yang et al., 2010; Broughton et al., 2013). The human amniotic membrane is formed from the epiblast on or around the eighth day after fertilization and before gastrulation, and constitutes the inner layer of the amnion surrounding the fetus (Akle et al., 1981). The human amniotic membrane has key functions in pregnancy and embryonic development; it has anti-inflammatory and immunological properties that act to suppress the immune response against the fetus, has multiple metabolic and transport functions and may contribute to the onset and progression of uterine contractility (Mamede et al., 2012). The cell populations within amniotic membrane demonstrate pluripotent properties, reflecting the origin of this membrane *in utero*, but also providing a rationale for clinical applications of these cells (Parolini et al., 2008).

**Figure 1:**
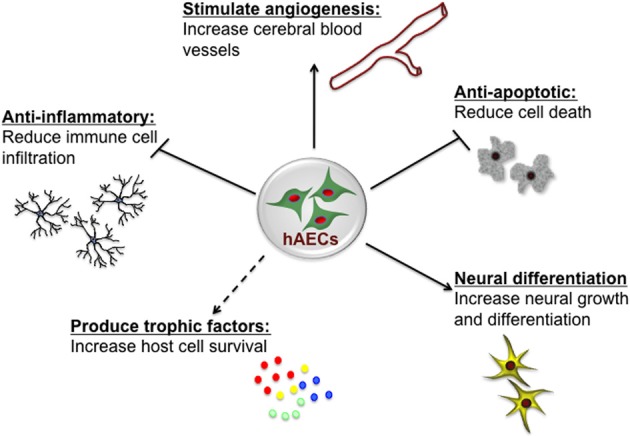
**Potential neuroprotective and neurorestorative mechanisms of human amnion epithelial cells (hAECs)**. hAECs have immunomodulatory properties which lead to a reduction of immune cell infiltration into the brain, thus reducing inflammation. They secrete various trophic and angiogenic factors which stimulate angiogenesis, neuronal differentiation and survival, and decrease apoptotic cell death, thus leading to improved functional outcomes.

The innermost layer of the human amnion is 8–12 μm thick and consists of a single layer of homogeneous cuboidal epithelial cells, so-called hAECs (Hebertson et al., 1986; Iwasaki et al., 2003). hAECs display many characteristics of both embryonic and pluripotent stem cells, with the potential to differentiate into a range of cell types (Ilancheran et al., 2007; Parolini et al., 2008). The amniotic fluid is also a source of cells with similar characteristics to hAECs. The heterogenous population of cells obtained from amniotic fluid at amniocentesis, have a diverse capacity to differentiate and, as for hAECs, may thus offer novel therapeutic potential (De Coppi et al., 2007). Both amniotic fluid-derived stem cells and hAECs are positive for CD117 (c-Kit) which is a transmembrane protein that functions as a tyrosine kinase receptor—a receptor that is also present on embryonic stem cells, primordial germ cells and somatic stem cells including neural crest cells—however, amniotic fluid-derived stem cells also appear distinct from hAECs on the basis of differences in many cell surface markers and in gene expression patterns assessed by transcriptional profiling (Hipp and Atala, 2006; Murphy et al., 2010). hAECs themselves have low immunogenicity (Bailo et al., 2004) and indeed, rather than eliciting an immune response, they can reduce such a response by inhibiting both innate and adaptive immune system cells (Li et al., 2005). The role of hAECs in immunomodulation appears multi-factorial, characterized by suppression of pro-inflammatory cytokines (Solomon et al., 2001), regulation of macrophage recruitment (Tan et al., 2013) and secretion of factors that inhibit the chemotactic activity of neutrophils and macrophages (Li et al., 2005). These properties of hAECs, as summarized in Figure 1, are very attractive when considering the potential for cell-based therapies for treatment of brain injury. Additionally amnion can be easily collected in a non-invasive manner from the placenta which is discarded in ~300,000 Australian births each year and hAECs can be isolated and stored for later clinical use, without the need for expansion (Murphy et al., 2010). In this respect, hAECs are readily available and their use avoids the ethical issues that remain one of the major limitations for the therapeutic use of embryonic stem cells (Yu et al., 2009).

## Perinatal hypoxic-ischemic brain injury and hAECs

Emerging evidence demonstrates the broad potential of hAECs in the treatment of brain injury. Naïve hAECs express specific neural marker genes, including neuro filament-M, myelin basic protein, microtubule-associated protein 2, and glial fibrillary acid protein (Mahmood et al., 2005), and cultured hAECs can be directed toward a neural lineage, differentiating into neurons and astrocytic cells (Sakuragawa et al., 1996; Ilancheran et al., 2007; Toda et al., 2007). In culture, naïve, undifferentiated hAECs are also reported to synthesize and secrete neurotransmitters including catecholamines, acetylcholine, and neurotrophic factors (Sakuragawa et al., 1997; Elwan et al., 1998; Kakishita et al., 2000).

The neural differentiation capacity and neurotrophic potential of hAECs has been examined in animal models of adult brain pathology. In a rat experimental model of Parkinson's disease [induced by 6-hydroxydopamine (6-OHDA) administration, and causing multiple lesions], transplanted hAECs prevented the death of dopaminergic neurons, mediated by the active secretion of neurotrophic factors (Kakishita et al., 2000). In ischemic stroke induced by middle cerebral artery occlusion in adults rats, intra-cerebral injection of hAECs improved functional recovery and decreased ischemic infarct volume, when compared with non-hAEC treated rats (Liu et al., 2008). Although intra-cerebral injections of such cells are unlikely to be promoted for treatment of either adult or neonatal brain injury, it has recently been shown that intravenous administration of amniotic fluid-derived stem cells promotes significant motor and cognitive recovery in rats subjected to ischemic stroke. Rats treated with amniotic fluid stem cells demonstrated a significant decrease in brain infarct volume and significantly increased endogenous cell proliferation within proliferative zones, compared to post-stroke rats that did not receive cell therapy (Tajiri et al., 2012). A recent literature review by Broughton and colleagues provide an overview of the mechanisms that contribute to ischemic stroke injury and explores the novel properties of hAECs and, to a lesser extent, amniotic fluid- derived stem cells, and the properties that make them appealing for stroke research (Broughton et al., 2013). Research in this field is still in its relative infancy, but certainly hAECs are considered a novel and promising cell-based therapy for ischemic and inflammatory adult brain injury.

In developing preterm fetal sheep, we have recently published data on the utility of hAECs to reduce organ injury and to elucidate the mechanisms by which hAECs have their effect (Vosdoganes et al., 2011; Hodges et al., 2012; Yawno et al., 2013). In particular, we have been interested in novel therapies to reduce or ameliorate inflammation-induced preterm lung and brain injury. hAECs (180 million cells) delivered at 0, 6, and 12 h relative to the time of inflammatory insult, mitigated structural lung injury in preterm fetal sheep *in utero* and were incorporated into lung tissue, albeit in relatively small numbers, where they differentiated into alveolar cells (Hodges et al., 2012). In response to intrauterine inflammation induced by intra-amniotic lipopolysaccharide, hAECs attenuated the fetal inflammatory response by decreasing the localized increase in pro-inflammatory cytokines within fetal lungs (Vosdoganes et al., 2011). In turn, we have also reported that, by attenuating the fetal inflammatory response, hAECs reduced fetal brain inflammation and reduced gray and white matter preterm brain injury (Yawno et al., 2013). The neuroprotective actions of hAECs in the fetal brain were likely via anti-inflammatory effects, whereby hAECs suppressed activated microglial cell upregulation within the developing brain, which we showed to be correlated with decreased cell death. A notable observation was that hAECs were able to ameliorate lipopolysaccharide-induced periventricular white matter injury, by preferentially protecting endogenous oligodendrocytes (Yawno et al., 2013). Our understanding of the principal actions of hAECs in the developing lung and brain have been extended in mice models of adult lung disease, where hAECs were shown to decrease macrophage infiltration in the lungs and to promote the reparative M2 “alternatively activated” phenotype of macrophages, with fewer M1 phenotype “classically activated” pro-inflammatory macrophages (Tan et al., 2013). In related experiments, hAECs have been shown to repair established adult lung damage; however, these reparative benefits were found to be dependent on the timing of hAEC administration. hAECs were most beneficial at day 14 and not when administered earlier at day 7, during a time of peak inflammation. This likely reflects the evolution of the immune and inflammatory environment post-insult and that hAECs are not only capable of preventing injury but are also capable of repair once the injury is already established (Vosdoganes et al., 2012). Further studies in models of adult lung disease have demonstrated that the actions of hAECs were independent of tissue engraftment and, most likely, were due to factors released by hAECs rather than through direct effects of the hAECs themselves (Tan et al., 2013). The fact that hAECs may be capable of repairing established injury make them exciting and intriguing candidates for repairing damage to the developing brain, which is known to be mediated by inflammatory events and is often only revealed once injury has occurred. Furthermore, the fact that they can act via anti-inflammatory actions that are independent of tissue engraftment provides a rationale for development of therapies using the reparative factors, *per se*.

## Umbilical cord blood stem cells

UCB contains a rich and diverse mixture of stem and progenitor cells that have the potential to generate a variety of cell types (Ali and Bahbahani, 2010). In 1989, Broxmeyer, Gluckman, and colleagues demonstrated that UCB can be used in clinical settings for stem cell transplantation (Broxmeyer et al., 1989; Gluckman et al., 1989). Since then, UCB has been used to treat nearly 80 diseases with over 25,000 transplants worldwide. UCB represents an abundant source of non-embryonic stem cells which are easily accessible with non-invasive collection of cells and no risk to the donor. Such cells are more immature than their bone marrow derived counterparts and hence display an impressive proliferative potential (Tiwari et al., 2012; Tursky et al., 2012) and have good viability after cells have been cryopreserved for later use (Pipes and Ablin, 2006; Slatter et al., 2006). UCB stem cells have high engraftment rates when used for replacement of haematopoietic stem cell populations, are relatively tolerant of HLA mismatches and thereby show low rates of graft-vs.-host disease, compared to bone marrow derived stem cells (Swijnenburg et al., 2005). They are rarely contaminated with latent viruses resulting in greater acceptance of UCB stem cells in comparison to those from bone marrow. UCB has been widely used for the treatment of various hematopoietic disorders (Kurtzberg, 1996; Locatelli et al., 1998) but, of relevance to this review, they have more recently been shown to induce regeneration in the central nervous system (Harris and Rogers, 2007; Herranz et al., 2010).

UCB is rich in hemapoietic stem/progenitor cells, regulatory T-lymphocytes (Tregs), monocytes, mesenchymal stem cells (MSCs), endothelial progenitor cells (EPCs), and stromal precursor cells (Pimentel-Coelho et al., 2012) and, consequently, holds promising potential for the treatment of neurological disorders. Indeed, a recent pre-clinical study has shown that UCB transplantation resulted in improved sensorimotor ability in a rat model of hypoxic ischemic brain injury (Geissler et al., 2011). To our knowledge, there are currently only a modest number of animal studies that have examined the effects UCB transplantation following hypoxic-ischemic injury, predominantly in newborn rats. These experiments using the Rice-Vannuci animal model (Rice et al., 1981) have reported positive brain results following UCB transplantation including decreased reactive gliosis (Wasielewski et al., 2012), increased tissue repair, cognitive improvements (de Paula et al., 2012), amelioration of injury-related effects in the primary somatosensory cortex (Geissler et al., 2011) and enhancement of endogenous neural stem cell proliferation via Hedgehog signaling (Wang et al., 2013b). These pre-clinical trials have not yet fully elucidated the mechanism underlying the beneficial effects of UCB transplantation, and there are several important questions that must be addressed before findings can be translated to the bedside. Nevertheless, autologous intravenous UCB transplantation has been shown to be safe and feasible in young children with acquired neurological disorders (Buzanska et al., 2006).

With respect to neonatal brain injury, UCB transplantation is emerging as a promising therapeutic approach for treatment of hypoxic-ischemic brain injury and ischemic stroke (Wang et al., 2012). There are now a number of human clinical trials taking place to examine the potential therapeutic benefits of undifferentiated cord blood cells for the treatment of established ischemic brain injury and established cerebral palsy. For Table 1 we identified these trials through the ClinicalTrials.gov database using the following search terms: UCB + newborn + ischemic brain injury OR + cerebral palsy. Although some trials have now been completed, there have been no peer-reviewed reports of them published as yet. It is imperative that the timing of the administration of the UCB with respect to the time of the injury (if known) is defined, as well as the optimal dose of UCB for transplantation. Furthermore, the contribution and beneficial effects of the different cell populations that are present in UCB need to be elucidated in order to determine adequate therapies that could lead to further improvement in neurological outcome, based on the clinical scenario. We will further discuss two different cell types found in cord blood, which independently show promise in pre-clinical trials.

**Table 1:** **Clinical trials being conducted around the world using umbilical cord blood in regenerative medicine therapies for the management of cerebral palsy and ischemic brain injury in the newborn**.

**Study title**	**Main objective**	**Institution**	**Treatment**	**Current status**	**Trial identifier**
A randomized study of autologous umbilical cord blood reinfusion in children with cerebral palsy	To determine the efficacy of a single intravenous infusion of autologous umbilical cord blood for the treatment of pediatric patients with spastic cerebral palsy.	Duke University, United States	Intravenous infusion. Autologous umbilical cord blood.	Currently recruiting	NCT01147653
			Timing: not specified. (children 12 months–6 years of age enrolled).		
Characterization of the cord blood stem cell in situation of neonatal asphyxia (NEOCORD)	To characterize cord blood stem cells of neonates with neonatal asphyxia and to compare them with those from healthy newborn.	Assistance publique Hopitaux de Marseille	*In vitro* characterization of the cord blood stem cell only.	Currently recruiting	NCT01284673
Allogenic umbilical cord blood and erythropoietin combination therapy for cerebral palsy	To determine efficacy of umbilical cord blood and erythropoietin combination therapy for children with cerebral palsy.	Sung Kwang Medical Foundation, Korea	Intravenous allogeneic umbilical cord blood infusion (total nucleated cells >3 × 10^∧^7/kg) in combination with erythropoietin given twice a week for 4 weeks.	Completed	NCT01193660
			Timing: up to 6 months after adverse event.		
Safety and effectiveness of cord blood stem cell infusion for the treatment of cerebral palsy in children	To test the safety and effectiveness of a cord blood infusion in children who have motor disability due to cerebral palsy. The subjects will be children whose parents have saved their infant's cord blood, who have non-progressive motor disability, and whose parents intend to have a cord blood infusion.	Georgia Health Sciences University, United States	Intravenous infusion of red-cell depleted, mononuclear cell enriched cord blood.	Currently recruiting	NCT01072370
			Timing: not specified. (children 1–12 years of age enrolled).		
Autologous stem cells in newborns with oxygen deprivation	To determine if the plasticity of autologous intravenous administration of cord blood stem cells would improve the clinical course of asphyxiated newborns.	Hospital Universitario, Monterrey, Mexico	Intravenous infusion of autologous cord and placental cord blood (CD34+ Cells).	Currently recruiting	NCT01506258
			Timing: within the first 48 h after birth.		
Umbilical cord blood therapy for cerebral palsy	To evaluate the efficacy of umbilical cord blood therapy for children with cerebral palsy.	Bundang CHA Hospital, Republic of Korea	Umbilical cord blood infusion intravenously or intraarterially under non-myeloablative immunosuppression.	Completed	NCT01528436
			Timing: not specified. (children 6 months–20 years of age enrolled).		
Umbilical cord blood therapy for children with cerebral palsy	To evaluate the efficacy of umbilical cord blood therapy for children with cerebral palsy.	Bundang CHA Hospital, Republic of Korea	Allogeneic umbilical cord blood infusion intravenously or intraarterially under non-myeloablative immunosuppression.	Ongoing, but NOT recruiting	NCT01639404
			Timing: not specified. (children 6 months–20 years of age enrolled).		
Autologous umbilical cord blood transfusion for preterm neonates	To test feasibility of collection, preparation and infusion of autologous umbilical cord blood in the first 14 days after birth if the baby is born premature <35 weeks of gestation.	Ain Shams University, Cairo, Egypt	Autologous intravenous cord blood transfusion.	Currently recruiting	NCT01121328
			Timing: within the first 14 postnatal day.		
Autologous cord blood cells for brain injury in term newborns	To test feasibility and safety of collection, preparation and infusion of autologous umbilical cord blood during the first 3 days of age if the baby is born with signs of brain injury.	National University Hospital, Singapore	Intravenous infusion of autologous cord blood.	Currently recruiting	NCT01649648
			Timing: 3 days post-birth.		
Cord blood for neonatal hypoxic-ischemic encephalopathy	To test feasibility of collection, preparation and infusion of a baby's own umbilical cord blood in the first 14 days after birth if the baby is born with signs of brain injury.	Duke University, United States	Intravenous infusions autologous volume reduced cord blood cells (up to 4 infusions).	Currently recruiting	NCT00593242
			Timing: first 18 postnatal days.		

Information obtained from ClinicalTrials.gov. September 2013.

## Endothelial progenitor cells (EPCs)

EPCs were originally identified as a population of stem cells in human peripheral blood and characterized by their expression of CD34, vascular endothelial receptor 2 (VEGFR-2), and CD133 markers (Asahara et al., 1997; Peichev et al., 2000). Subsequently, EPCs have been isolated from other sources, such as bone marrow, fetal liver, and UCB. Although no specific markers are currently available for EPCs, the early functional angioblast, located predominantly in the bone marrow are generally identified by three surface markers (CD133, CD34, and VEGF-2), while circulating EPCs (which have begun to mature into endothelial cells) are usually characterized by expression of endothelial markers such as VE-cadherin, endothelial nitric oxide synthase and von Willebrand factor (Hristov and Weber, 2004). EPCs are stem cells that are mobilized in response to acute hypoxia from bone marrow and released into the circulation. Endogenous EPCs act to maintain vascular integrity and homeostasis, and then, when required, they are able to mediate the response to, and the repair of, vascular injury. This reparative role is achieved by inducing endothelial cell repair and regeneration, and by promoting tissue neovascularization (Zhang et al., 2002). Experimental and human studies show that EPCs participate in neovascularization processes in ischemic organs, and hence their regulation could have therapeutic applications in vascular diseases (Zampetaki et al., 2008). In adult ischemic brain injury, such as occurs in stroke, transplanted EPCs home to the ischemic injury core, and promote cerebral neovascularization and neuron progenitor cell migration and survival (Zhang et al., 2002; Fan et al., 2010).

## EPCs, vascular integrity and repair

**Figure 2:**
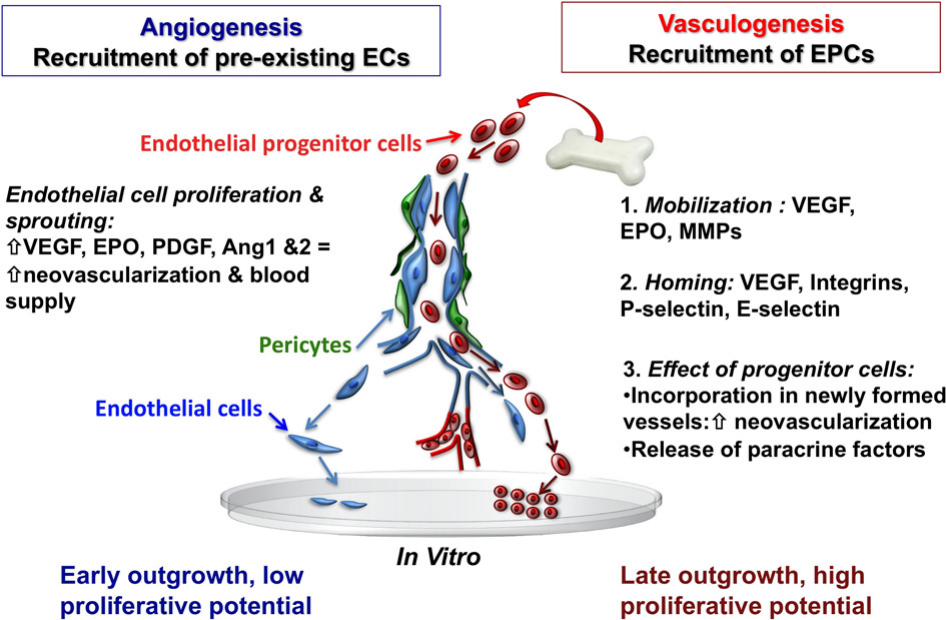
**Recruitment of endothelial cells from pre-existing vessel wall plays a critical role in the regulation of postnatal angiogenesis and vasculogenesis**. Mobilized bone marrow or umbilical cord blood-derived EPCs with high proliferative capacity may have the potential to migrate and incorporate into the injured tissue vascular bed. Abbreviations: EC, endothelial cells; EPCs, endothelial progenitor cells; VEGF, vascular endothelial growth factor; EPO, erythropoietin; MMPs, matrix metalloproteinases, PDGF, platelet derived growth factor; Ang 1and2, angiopoietin 1 and 2.

Maintaining the integrity of the vascular endothelial monolayer is extremely important, since it represents a barrier between the blood and subendothelial matrix proteins, but also restricts the infiltration of inflammatory cells, modulates vascular tone, and controls vascular smooth muscle proliferation (Zampetaki et al., 2008). The cerebrovascular system requires constant remodeling and dynamic adaptation of the vascular network in response to functional needs. In response to molecular cues that are initiated under conditions of hypoxia-ischemia, new blood vessel formation in adults has traditionally been understood to result primarily from angiogenesis, the process of local proliferation, migration, and remodeling of endothelial cells from a mature pre-existing endothelium (Red-Horse et al., 2007). This process is dependent upon an array growth factors, matrix metalloproteinases, cytokines, and integrins. To date, over 20 endogenous pro-angiogenic factors have been identified, including vascular endothelial growth factor (VEGF), platelet derived growth factor (PDGF), erythropoietin (EPO), and angiopoietin 1 and 2 (Ang-1&2; Figure 2). Angiogeneis begins with an increase in vascular permeability, followed by basement membrane and extracellular matrix degradation via matrix metalloproteinases (MMPs). Endothelial cells then initiate migration along newly deposited extracellular matrix tracts to form vessel sprouts. Finally, lumen-containing vessels are formed and integrated into the circulation (Red-Horse and Ferrara, 2006). From a therapeutic standpoint, the vasculature of the central nervous system has attracted attention since a number of human ailments such as stroke, retinopathy, cancer, and autoimmune disease are intimately associated with vasculopathy. Stem cells, most notably EPCs, show great potential as neurorestorative therapies as they target the basic components of the neurovascular unit (endothelial cells, astrocytes, pericytes and smooth muscle cells, neural stem cells, oligodendrocytes, and neurons) and the basic cellular elements that form the basement membrane.

With the discovery of EPCs, it was demonstrated that neovascularization after focal cerebral ischemia can also occur via vasculogenesis—the *de novo* process of blood vessel formation by differentiation and migration of EPCs in response to local cues (Figure 2). EPCs migrate locally to injured tissue and participate in neovacularization, regeneration of the injured endothelium, or wound healing by providing a proliferative pool of cells with the capacity to differentiate into mature vascular endothelial cells, and by secreting pro-angiogenic growth factors (Zampetaki et al., 2008). In this regard, EPCs are a potential tool for therapeutic angiogenesis. VEGF, MMP-9, and EPO are involved in the mobilization of EPCs into the circulation from the bone marrow (Zampetaki et al., 2008) and EPCs are recruited to new sites of vascularization, using cues that resemble an inflammatory response. The process of EPC homing to the site of injury includes detachment from the bone marrow niche, rolling into blood vessels, and traveling within the circulation. Once in the vicinity of an injured vessel, EPCs interact with the damaged endothelial monolayer in a similar way that leukocytes interact with activated endothelial cells. Thus, adhesion molecules previously identified as being involved in the inflammatory response, particularly leukocyte adhesion molecules (P-selectin, E-selectin, β 2-integrins) have now been identified as key regulators of EPC homing (Zampetaki et al., 2008). Systemic administration of umbilical cord blood-derived EPCs in adult mice results in significant protection against hypoxic-ischemic brain injury, with reduced infarct volume, decreased neutrophil infiltration, and increased focal blood flow at 48 h after ischemia (Ohta et al., 2006). Interestingly, this study also reported that circulating EPC levels were inversely correlated with cerebral infarction, but positively correlated with regional blood flow in hypoperfused areas of the brain after ischemia (Ohta et al., 2006), suggesting that EPCs may be a functional predictor of the wellbeing of cerebral vasculature. Additionally, the level of circulating EPCs and their migratory activity may serve as a marker for the risk of cardiovascular diseases, and even as a predictor of vascular function in many diseases (Vasa et al., 2001; Tepper et al., 2002; Baumhakel et al., 2006; Werner and Nickenig, 2006a,b).

## EPCs in pregnancy and the fetus

EPCs circulate at very low levels in adult human blood, and their frequency and function may be secondarily impaired in response to various complications (Vasa et al., 2001; Tepper et al., 2002; Baumhakel et al., 2006; Werner and Nickenig, 2006a,b). In contrast, EPCs are enriched in term UCB, and cord blood EPCs have been shown to proliferate faster and show enhanced vessel forming ability in comparison to adult-derived EPCs (Yoder et al., 2007; Au et al., 2008). Furthermore, the average number of cell progeny that can be derived from a single plate of cord blood EPCs is reportedly 100-fold greater when compared to those obtained from an adult, and can be serially re-plated and expanded exponentially in long-term culture (Ingram et al., 2004), suggesting that cord blood represents a rich source of EPCs, with the potential for storage in large quantities for clinical use.

In pregnancy, EPCs in the maternal circulation are present in the second trimester (Gussin et al., 2002), and the number of circulating EPCs increases gradually with gestational age (Sugawara et al., 2005). A more recent study measured circulating EPCs in cord blood at various stages of human gestation and found that in infants born at 24–28 weeks of gestational age, the cord blood yielded low levels of EPCs and high levels of MSCs, whereas at 33–36 weeks gestation the cord blood yielded higher levels of EPCs, equivalent to that found in term infants (Javed et al., 2008). This was confirmed in another study investigating circulating EPCs in preterm infants with bronchopulmonary dysplasia, finding that EPC levels were relatively low below 32 weeks gestation, and increased as pregnancy progressed. Additionally, they noted that extremely preterm infants with low levels of EPCs had an increased risk of developing bronchopulmonary dysplasia (Borghesi et al., 2009).

## Mesenchymal stem cells (MSCs)

MSCs are multi-potent cells with a strong capacity for self-renewal, which can be isolated from a variety of tissues, such as bone marrow, adipose tissue, umbilical cord, and UCB. MSCs have the ability to differentiate into a variety of cell types, depending on cues from their microenvironment. MSCs have been studied for use in neurologically related cell-based therapy in adult experimental animal models and in clinical trials of human brain disorders, such as Parkinson's and Huntington's disease, traumatic brain injury, and stroke (Berry et al., 2006; Hermann et al., 2010; Zilka et al., 2011; Kitada and Dezawa, 2012). MSCs home to areas of insult, where they promote tissue repair via secretion of soluble factors that enhance tissue regeneration, stimulate proliferation, migration, and differentiation and survival of endogenous local progenitor cells found in the microenviroment, as well as by decreasing inflammatory and immune reactions and apoptosis (Caplan and Dennis, 2006; Yi et al., 2012).

**Figure 3:**
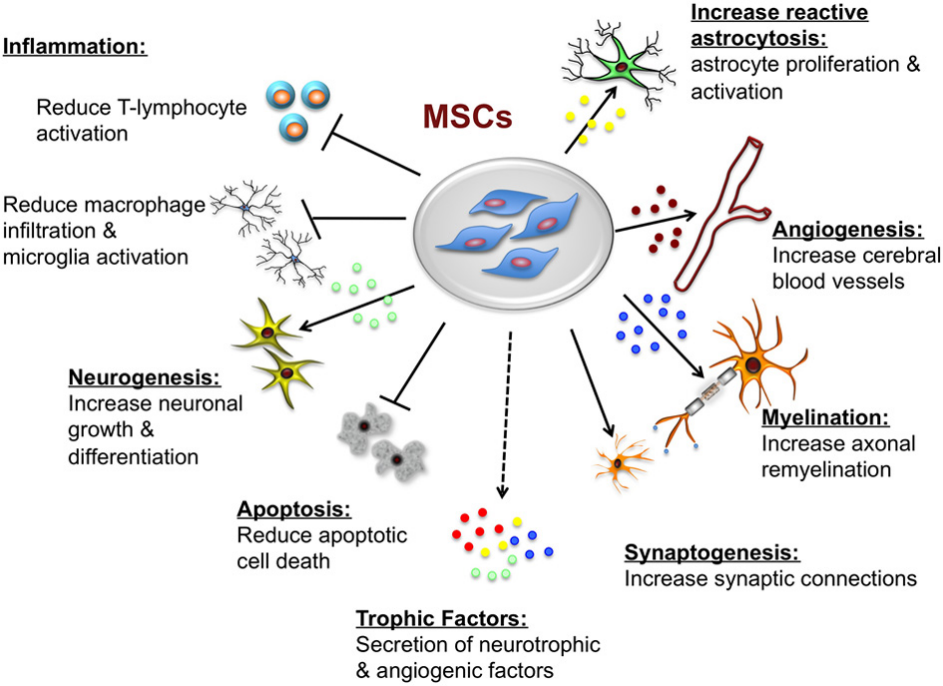
**Potential neuroprotective and neurorestorative effects of mesenchymal stem cells (MSCs)**. The beneficial actions of MSCs are mediated primarily by paracrine actions. MSCs secrete a number of neurotrophic and angiogenic factors that promote neuronal growth and differentiation, induce angiogenesis, neurogenesis and astroglial growth and activation, they promote synaptogenesis thus enhancing synaptic connections and axonal remyelination, decrease apoptosis, and decrease macrophage infiltration, and microglia and T-lymphocyte activation.

Transplanted MSCs augment host repair and tissue recovery, primarily through trophic support that appears to be mediated by direct and indirect actions. MSCs secrete both soluble (cytokines, growth factors) and insoluble (extracellular matrix proteins) factors that promote neural cell survival and regeneration (neurogenesis, angiogenesis, and synaptogenesis) through paracrine signaling (Seo and Cho, 2012). Other protective actions that are particular to the brain include remyelination (Akiyama et al., 2012), and inhibition of apoptosis and inflammation (Caplan and Dennis, 2006). The proposed neuroprotective effects of MSCs are summarized in Figure 3.

## Protective mechanisms of MSCs in brain injury

There is now solid preclinical data to demonstrate that MSC transplantation promotes functional recovery following experimental adult hypoxic ischemic brain injury or traumatic brain injury (Parr et al., 2007). The mechanisms by which MSCs regulate neural recovery and repair in brain tissue is now being extensively investigated, with a number of likely potential actions. In culture, MSCs can be directed toward, and may spontaneously generate, precursor and mature neuronal cells and cells of glial (astrocytic) lineage (Woodbury et al., 2000; Deng et al., 2006). When grafted into the lateral ventricle of the developing mouse brain, MSCs migrate over significant distances and preferentially differentiate into mature neurons and periventricular astrocytes (Deng et al., 2006), suggesting that MSCs are primed toward a neural fate. In addition to direct actions, MSCs secrete soluble factors capable of stimulating proliferation of neural stem cells *in vitro*, as well as increasing the expression of GFAP (a marker of mature astrocytes) (Galindo et al., 2011). Further studies confirm that MSCs activate neural stem cells or progenitor cells (Munoz et al., 2005, 2012), and are capable of undergoing expansion and differentiation into neurons, astrocytes, and oligodendrocytes both *in vitro* (Reynolds et al., 1992) and after transplantation *in vivo* (Alexanian et al., 2008; Kennea et al., 2013). In response to MSC transplantation after brain ischemia, there is an increase in new oligodendrocyte progenitor cells, mature oligodendrocytes, and myelin formation in the ischemic hemisphere (van Velthoven et al., 2011a). Transplantation of human MSCs into the injury penumbra of monkeys, one week following experimental cerebral ischemia, decreased apoptotic cell death, and the lesion volume (Li et al., 2010; Xin et al., 2010). In addition, Kim et al. (2010) showed that intravenous administration of human MSCs one day following traumatic brain injury in rats improved functional recovery and enhanced host cell survival by increasing pAkt and decreasing caspase-3 cleavage. Intravenous delivery of MSCs one day following experimental stroke in mice has also been shown to increase axon fiber density, synaptogenesis, and myelination (Xin et al., 2010). An important aspect of MSCs reparative role is the induction of trophic factors in response to injury cues; these include brain derived growth factor (BDNF), glial cell line-derived neurotrophic factor (GDNF), VEGF, fibroblast growth factor-2, and 7 (FGF-2, FGF-7), fibronectin, heparin binding-epidermal growth factor-like growth factor (HB-EGF), hepatocyte growth factor (HGF), interleukin-6 (IL-6), leukemia inhibitory factor (LIF), monocyte chemoattractant protein-1 (MCP-1), and PDGF (Li et al., 2002).

In addition to interacting directly with cells of the central nervous system, MSCs communicate with immune cells and have important immnosuppressive properties. MSCs suppress T- and B-cell proliferation, inhibit natural killer cell function, and modulate the secretory profile of microglia and macrophages (Le Blanc, 2003). Accordingly, treatment with MSCs is shown to decrease T-lymphocyte activity thereby exerting an immunoregulatory capacity (Fibbe et al., 2007; Gerdoni et al., 2007). In the ischemic brain, MSCs transplantation has been demonstrated to reduce the number of inflammatory cells (activated microglia and macrophages) (Kim et al., 2008). By reducing microglial expansion, MSC transplantation may reduce the inflammatory response, thus favoring the generation and integration of new neurons. MSC transplantation after acute traumatic brain injury in rats results in modulation of the inflammatory response by changing the expression of pro- and anti-inflammatory cytokines, along with modulation of serum levels of chemokines (Galindo et al., 2011). In a rat model of spinal cord injury, intravenously transplanted MSCs decrease the level of the pro-inflammatory cytokine IL-1β, reduced the number of activated microglia, and increased the level of anti-inflammatory IL-10 (Seo and Cho, 2012). Additionally, a recent spinal cord injury study showed that, similar to the actions described for hAECs, MSCs induced a shift in macrophages from M1 pro-inflammatory phenotype, to a M2 anti-inflammatory state, thereby promoting a protective and regenerative response (Busch et al., 2011).

Finally, as was described for the actions of EPCs, a principal aspect of regeneration in the injured brain is angiogenesis and vascular remodeling. In a rat model of stroke, intravenously injected MSCs were shown to migrate selectively toward ischemic areas of damaged brain to increase angiogenesis and improve functional recovery (Chen and Keating, 2001; Li et al., 2002). Evidence shows that these cells not only produce the appropriate cytokine milieu necessary to promote an angiogenic response, but they secrete pro-angiogenic factors such as VEGF, and regulate other trophic support for angiogenesis such as basic fibroblast growth factor (bFGF), BMP-2, angiogenin and IL-6. Interestingly, MSCs may also have the capacity to differentiate into endothelial cells (Miranville et al., 2004; Rehman et al., 2004; Potapova et al., 2007).

## MSCs in pregnancy and the fetus

MSCs can be detected in fetal blood from between 7 and 13 weeks gestation, with fetal MSCs being shown to have a characteristic morphology and immunophenotype (Campagnoli et al., 2001). Fetal MSCs are readily expandable *in vitro* and, as for adult bone marrow MSCs, they have the capacity to differentiate into a number of mesenchymal lineages (Pittenger et al., 1999; Reyes and Verfaillie, 2001; Devine, 2002). In the human fetus, circulating MSCs and EPCs appear at different gestational ages; at preterm 24–28 weeks gestational age, there is a significantly higher concentration of MSCs compared to EPCs within human UCB, which then switches in term gestation UCB. Accordingly, the concentration of MSCs decreases as human gestation proceeds to term, leading to a near absence of MSCs in 37–40 weeks UCB (Javed et al., 2008).

Despite the therapeutic potential that MSCs have demonstrated in adult brain injury, presently, there are only a handful of studies exploring the protective benefits of MSCs administered to the newborn. Studies arising from The Netherlands have guided the elucidation of the neuroprotective benefits of MSCs in the newborn brain; van Velthoven et al. (2011a) first demonstrated that bone-marrow derived MSCs administered in response to neonatal acute hypoxic ischemic brain damage improved functional outcome and reduced brain lesion volume. MSC-treated mice showed a decrease in the number of proliferating microglia in the lesioned hemisphere, decreased expression of proinflammatory cytokines in the ischemic hemisphere, and increased differentiation of neurons and oligodendrocytes (van Velthoven et al., 2011a,b). Most recently this group demonstrated that, in this acute newborn hypoxic-ischemic injury model, MSCs could be effectively administered intra-nasally and maintain their functional and structural neuroprotective benefits (Donega et al., 2013). Similarly, it has been reported that MSCs derived from human UCB and administered intra-cerebrally were able to ameliorate neonatal rat hypoxic-ischemic brain injury, with MSC-treated animals showing improved neurological score and differentiation of MSCs into astrocytes but not neurons (Xia et al., 2010). In a model of neonatal hyperoxic lung injury, intra-tracheal transplantation of human UCB-derived MSCs attenuated lung injury in neonatal rats, in a time-dependent manner, showing significant protection in the early inflammatory phase (Chang et al., 2013). To our knowledge, the only currently reported human study evaluating the therapeutic potential of MSCs on brain injury is a recent investigation in which umbilical cord MSCs were administered to a 5-years-old girl with established cerebral palsy. The patient was given 7 transplantations of MSCs over a 6-month period, and was followed for 28 months after the final transplantation. Specifically, the study reported a reduction of ambulation with tumble, with the patient being able to stand up by herself, as well as other improvements such as enhanced immunity, increased physical strength, and adjusted speech and comprehension (Wang et al., 2013a).

## Conclusions

Without question, stem cells therapies hold promise for the treatment of a wide range of neurological disorders, including for the treatment of perinatal brain injuries. Indeed the potential and great hope placed in stem cells likely underlies the new trend of “stem cell tourism.” But, as indicated in this review, there remains many and varied unresolved or not yet addressed important questions about the timing, appropriate cell types, treatment strategies, and appropriate outcomes that must still be addressed before such cells can be safely used in clinical translation studies. Have we yet adequately explored the long-term safety of cord blood or hAECs administered to babies or children? Over the past several years experimental animal studies have provided essential insight into the efficacy and potential mechanisms of stem cell therapies to protect and repair the adult brain, yet there is still a relative lack of translational research using experimental models of fetal and neonatal brain injury. Despite this, and as shown in Table 1, there are a number of clinical trials being conducted throughout the world investigating the effects of UCB on acquired brain injury in the newborn. An in depth examination of the safety and efficacy of stem cells in experimental models of fetal and neonatal brain injury is urgently needed in order to advance stem cell therapy from the laboratory to the clinic. More importantly, there is currently no consensus regarding the optimal dose, mode of delivery and the timing of stem cell treatment, which are likely to result in the greatest improvement of neurological outcome, thus highlighting the importance of future pre-clinical studies will well-defined outcomes.

### Conflict of interest statement

The authors declare that the research was conducted in the absence of any commercial or financial relationships that could be construed as a potential conflict of interest.
